# Supplementary material for: Ontologizing health systems data at scale: making translational discovery a reality
Source: NPJ Digit Med. 2023 May 19;6:89. doi: 10.1038/s41746-023-00830-x (PMC10196319; doi:10.1038/s41746-023-00830-x)
Supplement: Supplementary file 1 — Supplementary Material [file 41746_2023_830_MOESM1_ESM.pdf]

Ontologizing Health Systems Data at Scale: Making Translational Discovery a Reality

SUPPLEMENTARY MATERIAL

Tiffany J. Callahan, Adrianne L. Stefanski, Jordan M. Wyrwa, Chenjie Zeng, Anna Ostropolets, Juan M. Banda, William A. Baumgartner Jr., Richard D. Boyce, Elena Casiraghi, Ben D. Coleman, Janine H. Collins, Sara J. Deakyne Davies, James A. Feinstein, Asiyah Y. Lin, Blake Martin, Nicolas A. Matentzoglu, Daniella Meeker, Justin Reese, Jessica Sinclair, Sanya B. Taneja, Katy E. Trinkley, Nicole A. Vasilevsky, Andrew E. Williams, Xingmin A. Zhang, Joshua C. Denny, Patrick B. Ryan, George Hripcsak, Tellen D. Bennett, Melissa A. Haendel, Peter N. Robinson, Lawrence E. Hunter, Michael G. Kahn

Table of Contents

Supplementary Table 1: Paper Acronyms and Concept Definitions. **1**

Supplementary Table 2: OMOP2OBO Mapping Algorithm Resources. **2**

Supplementary Table 3: Clinical Data Used to Develop and Validate the OMOP2OBO Mappings. **4**

Supplementary Figure 1: Available Mapping Metadata by OBO Foundry Ontology. **5**

Supplementary Table 4: OMOP2OBO Mapping Categories. **6**

Supplementary Table 5: OMOP2OBO Condition Concept Mapping Results. **8**

Supplementary Table 6: OMOP2OBO Drug Ingredient Concept Mapping Results. **9**

Supplementary Table 7: OMOP2OBO Measurement Concept Mapping Results. **10**

Supplementary Figure 2: Concept Similarity Scores by OMOP Domain and OBO Foundry Ontology. **11**

Supplementary Figure 3: Overview of the OMOP2OBO Condition Concepts in the OHDSI Concept Prevalence Data by Coverage Status. **12**

Supplementary Figure 4: Frequency of OMOP2OBO Condition Concepts in the OHDSI Concept Prevalence Data by OBO Foundry Ontology and Data Wave. **13**

Supplementary Figure 5: Overview of the OMOP2OBO Drug Ingredient Concepts in the OHDSI Concept Prevalence Data by Coverage Status. **14**

Supplementary Figure 6: Frequency of OMOP2OBO Drug Ingredient Concepts in the OHDSI Concept Prevalence Data by OBO Foundry Ontology and Data Wave. **15**

Supplementary Figure 7: Overview of the OMOP2OBO Measurement Concepts in the OHDSI Concept Prevalence Data by Coverage Status. **16**

Supplementary Figure 8: Frequency of OMOP2OBO Measurement Concepts in the OHDSI Concept Prevalence Data by OBO Foundry Ontology and Data Wave. **17**

Supplementary Figure 9: Standardized Phenotype Risk Scores (PheRS) by Disease for Cases and Controls. **18**

Supplementary Table 8: Descriptive Statistics by Disease for Cases and Controls. **19**

Supplementary Table 1: Paper Acronyms and Concept Definitions.

Term	Definition
Acronyms	
ACMG	American College of Medical Genetics and Genomics
AoU	All of Us Research Program
BoW	Bag-of-words
CDM	Common Data Model
CHCO	Children’s Hospital of Colorado
ChEBI	Chemical Entities of Biological Interest
CL	Cell Ontology
CUI	Concept Unique Identifier
EHR	Electronic Health Record
eMERGE	Electronic Medical Records and Genomics
FBN1	Fibrillin 1
HPO	Human Phenotype Ontology
ICD	International Classification of Diseases
LOINC	Logical Observation Identifiers, Names and Codes
MEN1	Menin 1
Mondo	Mondo Disease Ontology
NCBITaxon	National Center for Biotechnology Information Organismal Taxonomy
NF2	Moesin-Ezrin-Radixin Like (MERLIN) Tumor
OHDSI	Observational Health Data Sciences and Informatics
OBO	Open Biological and Biomedical Ontology
OMIM	Online Mendelian Inheritance in Man
OMOP	Observational Medical Outcomes Partnership
PEDSnet	National Pediatric Learning Health System
PheRS	Phenotype Risk Score
PRO	Protein Ontology
RET	Ret Proto-Oncogene
SDHAF2	Succinate Dehydrogenase Complex Assembly Factor 2
SDHB	Succinate Dehydrogenase Complex Subunit B
SDHC	Succinate Dehydrogenase Complex Subunit C
SNOMED-CT	Systematized Nomenclature of Medicine -- Clinical Terms
TF-IDF	Term frequency-inverse document frequency
TGFBR1	Transforming Growth Factor Beta Receptor 1
TSC1	Tuberous Sclerosis Complex Subunit 1
TSC2	Tuberous Sclerosis Complex Subunit 2
Uberon	Uber-Anatomy Ontology
UMLS	Unified Medical Language System
VO	Vaccine Ontology

Term	Definition
Concepts	
Concepts Used in Clinical Practice	Data Wave 1; All standard OMOP concepts used at least once in clinical practice
Concepts Not Used in Clinical Practice	Data Wave 2; All standard OMOP concepts not used in clinical practice
OMOP Standard Condition Occurrence Vocabulary	SnomedCT Release 20180131
OMOP Standard Drug Exposure Ingredient Vocabulary	RxNorm Full 20180507
OMOP Standard Measurement Vocabulary	LOINC 2.64
OBO Foundry Ontologies mapped to OMOP Conditions	HPO, Mondo
OBO Foundry Ontologies mapped to OMOP Drug Ingredients	ChEBI, NCBITaxon, PRO, VO
OBO Foundry Ontologies mapped to OMOP Measurements	ChEBI, CL, HPO, NCBITaxon, PRO, Uberon

Supplementary Table 2: OMOP2OBO Mapping Algorithm Resources.

Resource	URL
OMOP2OBO Resources	
PyPI Package	https://pypi.org/project/omop2obo
GitHub Repository	https://github.com/callahantiff/OMOP2OBO
Project Wiki	https://github.com/callahantiff/OMOP2OBO/wiki
Mapping Dashboard	http://tiffanycallahan.com/OMOP2OBO_Dashboard
Zenodo Community	https://zenodo.org/communities/omop2obo
Condition Occurrence Mappings	https://doi.org/10.5281/zenodo.6774363
Drug Exposure Ingredient Mappings	https://doi.org/10.5281/zenodo.6774401
Measurement Mappings	https://doi.org/10.5281/zenodo.6774443
Accuracy Evaluation	https://github.com/callahantiff/OMOP2OBO/wiki/Accuracy
Generalizability Evaluation	https://github.com/callahantiff/OMOP2OBO/wiki/Generalizability
Mapping Resources	
OMOP CDM V5.3	https://ohdsi.github.io/CommonDataModel/cdm53.html
OHDSI Athena	https://athena.ohdsi.org
UMLS 2020AA Release Date	https://www.nlm.nih.gov/research/umls/licensedcontent/umlsarchives04.html#2020AA
LOINC2HPO Annotations	https://github.com/monarch-initiative/loinc2hpo/annotations.tsv
OHDSI Concept Prevalence Study	https://github.com/OHDSI/StudyProtocolSandbox/tree/master/ConceptPrevalence
OBO Foundry Ontologies	
ChEBI	http://purl.obolibrary.org/obo/chebi.owl
CL	http://purl.obolibrary.org/obo/cl.owl
HPO	http://purl.obolibrary.org/obo/hp.owl
Mondo	http://purl.obolibrary.org/obo/mondo.owl
NCBITaxon	http://purl.obolibrary.org/obo/ncbitaxon.owl
PRO	http://purl.obolibrary.org/obo/pr.owl
Uberon	http://purl.obolibrary.org/obo/uberon/ext.owl
VO	http://purl.obolibrary.org/obo/vo.owl
Project and Analysis Notebooks	
[a]OMOP2OBO	[b]OMOP2OBO/blob/master/omop2obo_notebook.ipynb
Mapping Analysis	[b]OMOP2OBO/blob/master/resources/analyses/omop2obo_manuscript_analyses.ipynb
Mapping Evaluation	[b]OMOP2OBO/blob/master/resources/analyses/omop2obo_mapping_validation.ipynb

[a]This Jupyter Notebook serves the same purpose as main.py but provides users with a more interactive interface to use when running the algorithm.

[b]Primary OMOP2OBO Github: https://github.com/callahantiff/OMOP2OBO.

Acronyms: ChEBI (Chemical Entities of Biological Interest); CL (Cell Ontology); HPO (Human Phenotype Ontology); Mondo (Mondo Disease Ontology); NCBITaxon (National Center for Biotechnology Information Taxon Ontology); OBO (Open Biological and Biomedical Ontology); OMOP (Observational Medical Outcomes Partnership); PRO (Protein Ontology); Uberon (Uber-Anatomy Ontology); VO (Vaccine Ontology).

Supplementary Table 3: Clinical Data Used to Develop and Validate the OMOP2OBO Mappings.

Data Source	Description	Use
CHCO OMOP Database	The CHCO pediatric OMOP database is a de-identified data repository that allows for the utilization of clinical pediatric information captured in electronic medical records. The database was created in October 2018, contains over 6 million patients, and is stored within University of Colorado Anschutz Medical Campus' Health Data Compass HIPAA Google Cloud-based infrastructure. The data conform to the structure defined by PEDSnet OMOP CDM v3.0, which is an adaptation of the OMOP CDM version 5.0. Use of these data was approved by the Colorado Multiple Institutional Review Board (#15-0445).<br><br>See GitHub[a] for more information: https://github.com/HealthDataCompass/CHCODeID	Mapping Development
OHDSI Concept Prevalence Data	The Concept Prevalence Study was conducted in order to examine patterns of OMOP standard concept use across several study sites within the OHDSI network. The data set includes OMOP standard concepts, OMOP domain, and record-level frequencies for each standard concept by study site. All study sites that contained data for standard OMOP condition, drug exposure ingredient, and measurement concepts were eligible for use in the current work (n=22 sites). These data were supplemented to include data from two additional academic medical centers. The 24 Study sites are listed below.<br><br>Study Sites: (1) Ajou University Database; (2) IQVIA US Ambulatory Electronic Medical Record; (3) IQVIA Longitudinal Patient Data Australia; (4) IQVIA Disease Analyzer France; (5) IQVIA Disease Analyzer Germany; (6) The Healthcare Cost and Utilization Project Nationwide Inpatient Sample; (7) IQVIA US Hospital Charge Data Master; (8) IBM MarketScan Commercial Database; (9) IBM MarketScan Multi-State Medicaid Database; (10) IBM MarketScan Medicare Supplemental Database; (11) Japan Medical Data Center database; (12) Medical Information Mart for Intensive Care III; (13) Korea National Health Insurance Service/National Sample Cohort; (14) Optum De-Identified Clinformatics Data-Mart-Database—Date of Death; (15) Optum De-Identified Clinformatics Data-Mart-Database—Socio-Economic Status; (16) Optum De-identified Electronic Health Record Dataset; (17) IQVIA US LRxDx Open Claims; (18) Premier Healthcare Database; (19) University of Southern California PScanner; (20) Stanford Medicine Research Data Repository; (21) Tufts Medical Center Database; (22) University of Colorado Anschutz Medical Campus Health Group; (23) Australian Electronic Practice-based Research Network; (24) Columbia University Medical Center Database.<br><br>See GitHub for more information: https://github.com/ohdsi-studies/ConceptPrevalence	Mapping Validation<br>*Generalization*
AoU Data	The National Institutes of Health's All of Us Research Program is an initiative tasked with gathering data from at least one million United States citizens with the goal of creating a diverse health resource to support biomedical research and precision medicine. The All of Us Research Hub contains data from over 630 sites on more than 528,000 participants. Data include electronic health records, biological and genetics samples, physical measurements and wearable data, and survey data. The All of Us Research Program would not be possible without the partnership of its participants. The current work utilized data from the version 6 build.<br><br>See the All of Us Research Hub for more information: https://www.researchallofus.org	Mapping Validation<br>*Clinical Utility*

[a]This is a private repository, please contact the authors for access and to obtain additional information.

Acronyms: AoU (AllOfUs); CDM (common data model); CHCO (Children's Hospital Colorado); HIPAA (Health Insurance Portability and Accountability Act); OHDSI (Observational Health Data Sciences and Informatics); OMOP (Observational Medical Outcomes Partnership; PEDSnet (National Pediatric Learning Health System).

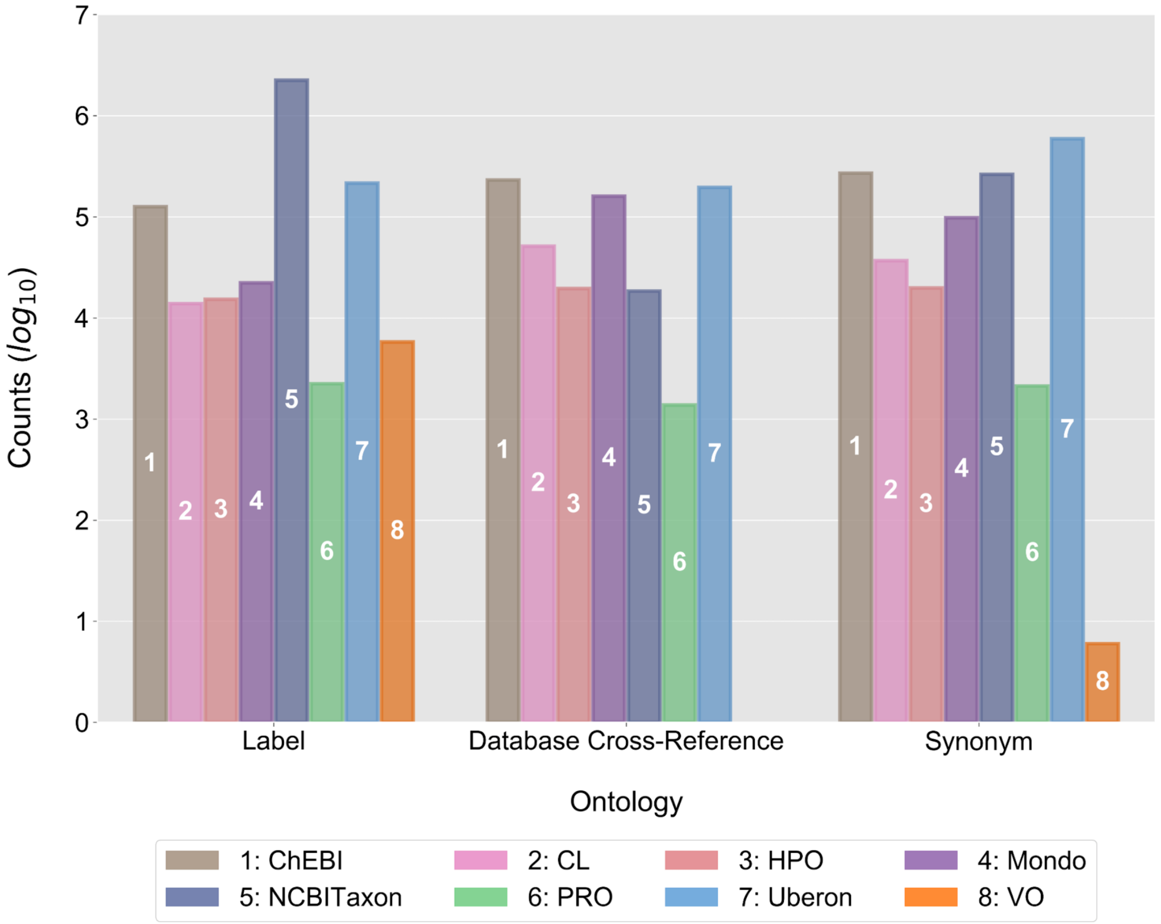

Supplementary Figure 1: Available Mapping Metadata by OBO Foundry Ontology.

This figure provides a visual illustration of the counts, in log 10 scale, of labels, database cross-references, and synonyms available for mapping by Open Biological and Biomedical Ontology (OBO) Foundry ontology. The labels on the bars are numbers which correspond to the ontologies: (1) ChEBI (Chemical Entities of Biological Interest); (2) CL (Cell Ontology); (3) HPO (Human Phenotype Ontology); (4) Mondo (Mondo Disease Ontology); (5) NCBITaxon (National Center for Biotechnology Information Taxon Ontology); (6) PRO (Protein Ontology); (7) Uberon (Uber-Anatomy Ontology); and (8) VO (Vaccine Ontology).

Supplementary Table 4: OMOP2OBO Mapping Categories.

Mapping Category	Definition
Automatic One-to-One Concept	**Definition:** A one-to-one mapping that is automatically generated at the concept-level through exact string mappings to labels/synonyms or exact mappings between codes.<br>**Example:**<br>- `OMOP:22945` (Horizontal overbite)<br>- `HP:0011095` (Overjet)<br>This mapping was created through an exact string mapping on "overjet", which is the HP concept label and an OMOP concept synonym. This mapping is also supported through exact mappings between database cross-references to SNOMED-CT 70305005 and UMLS C0596028.
Automatic One-to-One Ancestor	**Definition:** A one-to-one mapping that is automatically generated for a concept's ancestor through exact string mappings to labels/synonyms or exact mappings between codes.<br>**Example:**<br>- `OMOP:22722` (Accessory salivary gland)<br>- `HP:0010286` (abnormal salivary gland morphology)<br>This mapping was created through exact mappings to one of the OMOP concept's ancestors on the database cross-references to SNOMED-CT 10890000 and UMLS C0036093.
Automatic One-to-Many Concept	**Definition:** A one-to-many mapping that is automatically generated at the concept-level through exact string mappings to labels/synonyms or exact mappings between codes. For release 1.0, one-to-many mappings indicate that one OMOP concept was mapped to one or more OBO Foundry ontology concepts.<br>**Example:**<br>- `OMOP:78854` (Osteopoikilosis)<br>- `MONDO:0001414` (Osteopoikilosis) AND `MONDO:0008157` (Duschke-Ollendorff Syndrome)<br>This mapping was created through 2 exact string mappings on "osteopoikilosis", which is a Mondo concept exact synonym and an OMOP concept label and synonym and "duschke-ollendorff syndrome", which is a Mondo concept exact synonym and label and an OMOP concept synonym. This mapping is also supported through exact mappings between database cross-references to SNOMED-CT 9147009.
Automatic One-to-Many Ancestor	**Definition:** A one-to-many mapping that is automatically generated for a concept's ancestor through exact string mappings to labels or synonyms or exact mappings between codes. For release 1.0, one-to-many mappings indicate that one OMOP concept was mapped to one or more OBO Foundry ontology concepts.<br>**Example:**<br>- `OMOP:74185` (Open fracture of cuboid bone of foot)<br>- `MONDO:0005315` (bone fracture) AND `MONDO:0044989` (foot disease)<br>This mapping was created through 3 exact string mappings on "fracture", "fracture of bone", and "disorder of foot", which are all Mondo exact synonyms and labels of the OMOP concept's ancestors. This mapping is also supported by exact mappings to one or more of the OMOP concept's ancestors on the database cross-references to SNOMED-CT 125605004 and 118932009.
Manual One-to-One Concept	**Definition:** A one-to-one mapping that is manually generated at the concept-level and usually requires the use of external resources.<br>**Example:**<br>- `OMOP:4070954` (Mesiodens)<br>- `MONDO:0008533` (Teeth, supernumeracy)<br>This mapping was manually created through external evidence from a PubMed article, which stated "Mesiodens is a supernumerary tooth present in the midline between the two central incisors" (`PMID:21998774`).
Manual One-to-Many Concept	**Definition:** A one-to-many mapping that is manually generated at the concept-level and usually requires the use of external resources. For release 1.0, one-to-many mappings indicate that one OMOP concept was mapped to one or more OBO Foundry ontology concepts.<br>**Example:**<br>- `OMOP:439140` (Neonatal polycythemia)<br>- `HP:0003623` (Neonatal onset) AND `HP:0001901` (Polycythemia)<br>This mapping was created through an exact string mappings on "erythrocytosis", which is a HP concept exact synonym and a OMOP concept ancestor label. This mapping is also supported through exact mappings between database cross-references to SNOMED-CT 127062003 and UMLS C1527405 and C0032461.

<table>
<tr><th>Mapping Category</th><th>Definition</th></tr>
<tr><td>Cosine Similarity One-to-One Concept</td><td>Definition: A one-to-one mapping that is automatically generated at the concept-level using cosine similarity scores. For release 1.0, the cosine similarity scores were applied to concept embeddings learned from a Bag-of-Words model with TF-IDF, which was applied to all available labels and synonyms at the concept- and ancestor-level.
Example:
- OMOP:4147326 (Sore throat symptom)
- HP:0033050 (Throat pain)
This mapping received a cosine similarity score of 0.66.</td></tr>
<tr><td>Unmapped</td><td>This concept is used when no suitable mapping is possible, for concepts which have not yet been mapped, and for concepts which are purposefully not mapped.
Examples:
No Suitable Mondo Mapping
- OMOP:4235440 (Genetic alleles)
Not Yet Mapped to HP or Mondo
- OMOP:4174055 (Athetoid paralysis)
Purposefully Not Mapped to HP or Mondo
- OMOP:432499 (Mechanical complication due to coronary bypass graft) → Complication
- OMOP:432498 (Burn of axilla) → Injury
- OMOP:4056963 (Patient on self-medication) → Finding</td></tr>
</table>

Acronyms: HP (Human Phenotype Ontology); Mondo (Mondo Disease Ontology); OMOP (Observational Medical Outcomes Partnership); PMID (PubMed Identifier); SNOMED-CT (Systematized Nomenclature of Medicine -- Clinical Terms); UMLS (Unified Medical Language System).

Supplementary Table 5: OMOP2OBO Condition Concept Mapping Results.

	HPO		Mondo	
Concepts Used in Practice	Yes	No	Yes	No
Mapping Category				
Automatic One-to-One Concept	3,601	1,166	4,836	4,261
Automatic One-to-One Ancestor	3,154	10,440	5,962	2,949
Automatic One-to-Many Concept	125	25	632	253
Automatic One-to-Many Ancestor	1,138	36,947	4,482	35,742
Cosine Similarity One-to-One Concept	994	380	553	114
Manual One-to-One Concept	5,119	0	755	0
Manual One-to-Many Concept	10,328	0	2,835	0
Total Mapped Concepts	**24,459**	**48,958**	**20,055**	**43,319**
Mapping Evidence				
Database Cross-References	38,473	279,236	52,430	339,195
Synonyms	10,169	42,191	67,381	85,130
Labels	19,343	97,920	75,795	113,562
Cosine Similarity	11,955	15,825	12,789	114
Biocuration	15,447	0	3,590	0
Total Mapping Evidence	**95,387**	**435,172**	**211,985**	**538,001**
Unmapped				
[a]None	50	20,771	84	5,118
Injury	3,323	10,733	3,323	10,733
Carrier Status	23	0	22	0
Complication	906	128	906	128
Finding	368	0	4,739	21,292
Total Unmapped Concepts	**4,670**	**31,632**	**9,074**	**37,271**

The mapping category is constructed by combining the following elements: (1) the approach used to create it (i.e., "automatic", "manual", or "cosine similarity"), (2) cardinality (i.e., one-to-one or one-to-many), and (3) level (i.e., concept or ancestor).

[a]The unmapped "None" category for Concepts Not Used in Practice includes concepts that have not yet been mapped. For Concepts Used in Practice, "None" indicates concepts that were unable to be mapped to an Open Biological and Biomedical Ontology (OBO) Foundry ontology concept.

Acronyms: HPO (Human Phenotype); Mondo (Mondo Disease Ontology).

Supplementary Table 6: OMOP2OBO Drug Ingredient Concept Mapping Results.

	ChEBI		PRO		VO		NCBITaxon	
Concepts Used in Practice	Yes	No	Yes	No	Yes	No	Yes	No
Mapping Category								
Automatic One-to-One Concept	959	2,192	1	42	90	18	20	135
Automatic One-to-One Ancestor	15	130	1	19	0	4	3	14
Automatic One-to-Many Concept	235	169	0	1	0	0	0	1
Automatic One-to-Many Ancestor	60	149	2	0	2	0	2	1
Cosine Similarity One-to-One Concept	31	78	8	10	3	14	136	4,105
Manual One-to-One Concept	321	0	157	0	21	0	230	0
Manual One-to-Many Concept	72	0	8	0	2	0	14	0
Total Mapped Concepts	**1,693**	**2,718**	**177**	**72**	**118**	**36**	**405**	**4,256**
Mapping Evidence								
Database Cross-References	954	759	0	0	0	0	0	0
Synonyms	4,565	7,732	4	94	90	18	40	199
Labels	5,573	9,676	8	132	276	58	52	391
Cosine Similarity	1,350	2,562	9	54	96	32	160	4,241
Biocuration	393	0	165	0	23	0	244	0
Total Mapping Evidence	**12,835**	**20,729**	**186**	**280**	**485**	**108**	**496**	**4,831**
Unmapped								
[a]None	0	7,392	1,516	10,038	1,575	10,074	1,288	5,854
Total Unmapped Concepts	**0**	**7,392**	**1,516**	**10,038**	**1,575**	**10,074**	**1,288**	**5,854**

The mapping category is constructed by combining the following elements: (1) the approach used to create it (i.e., “automatic”, “manual”, or “cosine similarity”), (2) cardinality (i.e., one-to-one or one-to-many), and (3) level (i.e., concept or ancestor).

[a]The unmapped “None” category for Concepts Not Used in Practice includes concepts that have not yet been mapped. For Concepts Used in Practice, “None” indicates concepts that were unable to be mapped to an Open Biological and Biomedical Ontology (OBO) Foundry ontology concept.

Acronyms: ChEBI (Chemical Entities of Biological Interest); PRO (Protein Ontology); VO (Vaccine Ontology); NCBITaxon (National Center for Biotechnology Information Taxon Ontology).

Supplementary Table 7: OMOP2OBO Measurement Concept Mapping Results.

	HPO		Uberon		NCBITaxon		PRO		ChEBI		CL	
Concepts Used in Practice	Yes	No	Yes	No	Yes	No	Yes	No	Yes	No	Yes	No
Mapping Category												
Automatic One-to-One Concept	17	3	1,793	3,589	320	444	44	12	264	515	182	186
Automatic One-to-One Ancestor	23	20	592	593	181	351	9	6	1,380	1,924	14	0
Automatic One-to-Many Concept	0	0	10	0	0	0	0	0	0	0	46	24
Automatic One-to-Many Ancestor	0	0	2	0	0	0	0	0	29	3	3	0
Cosine Similarity One-to-One Concept	108	5	50	92	44	106	103	29	102	374	82	20
Manual One-to-One Concept	3,902	6,761	406	462	2,300	4,452	1,267	2,996	1,377	2,409	319	184
Manual One-to-Many Concept	37	12	1,234	2,065	5	454	149	189	337	1,190	33	21
Total Mapped Concepts	**4,087**	**6,801**	**4,087**	**6,801**	**2,850**	**5,807**	**1,572**	**3,232**	**3,489**	**6,415**	**679**	**435**
Mapping Evidence												
Database Cross-References	7	0	6	26	0	0	0	0	409	935	261	145
Synonyms	12	4	5,232	8,308	465	1,627	73	24	2,832	6,166	486	414
Labels	28	24	1,637	1,242	307	458	29	14	3,045	5,712	296	227
Cosine Similarity	234	128	699	553	484	827	159	61	1,482	2,044	296	231
Biocuration	3,939	6,773	1,640	2,527	2,305	4,906	1,416	3,185	1,714	3,599	352	205
Total Mapping Evidence	**4,220**	**6,929**	**9,214**	**12,656**	**3,561**	**7,818**	**1,677**	**3,284**	**9,482**	**18,456**	**1,691**	**1,222**
Unmapped												
[a]None	13	0	13	0	1,250	994	2,528	3,569	611	386	3,421	6,366
Not Mapped Test Type	108	3	108	3	108	3	108	3	108	3	108	3
Unspecified Sample	217	40	217	40	217	40	217	40	217	40	217	40
Total Unmapped Concepts	**338**	**43**	**338**	**43**	**1,575**	**1,037**	**2,853**	**3,612**	**936**	**429**	**3,746**	**6,409**

The mapping category is constructed by combining the following elements: (1) the approach used to create it (i.e., "automatic", "manual", or "cosine similarity"), (2) cardinality (i.e., one-to-one or one-to-many), and (3) level (i.e., concept or ancestor).

[a]The unmapped "None" category for Concepts Not Used in Practice includes concepts that have not yet been mapped. For Concepts Used in Practice, "None" indicates concepts that were unable to be mapped to an Open Biological and Biomedical Ontology (OBO) Foundry ontology concept.

Acronyms: HPO (Human Phenotype Ontology); Uberon (Uber-Anatomy Ontology); NCBITaxon (National Center for Biotechnology Information Taxon Ontology); PRO (Protein Ontology); ChEBI (Chemical Entities of Biological Interest); CL (Cell Ontology).

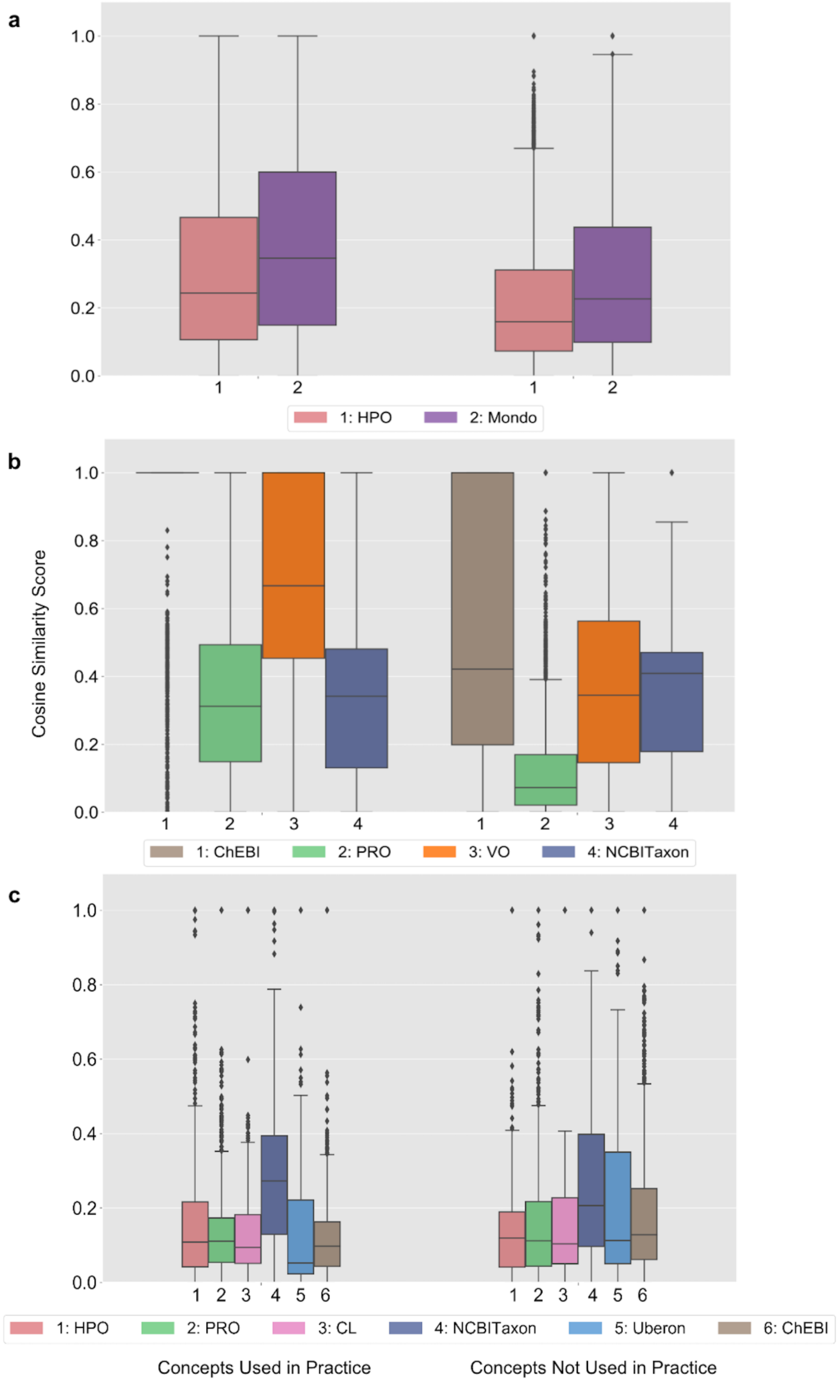

Supplementary Figure 2: Concept Similarity Scores by OMOP Domain and OBO Foundry Ontology.

The figure presents the distribution of cosine similarity scores by Open Biological and Biomedical Ontology (OBO) Foundry ontology and data wave (Concepts Used in Practice [all concepts associated with at least one patient and/or visit in the Children's Hospital of Colorado OMOP Database] and Concepts Not Used in Practice [all concepts not used in clinical practice]) for three Observational Medical Outcomes Partnership (OMOP) domains: (**A**) Conditions, (**B**) Drugs, and (**C**) Measurements. In each boxplot, the box extends from the first to third quartile of the data with a center line used to indicate the median. Whiskers extend from each box by 1.5x the interquartile range and outliers that extend past the whiskers shown as dots. The x-axis labels are numbers which correspond to the ontologies within each domain from top to bottom: Conditions (1: HPO, 2: Mondo); Drug Ingredients (1: ChEBI, 2: PRO, 3: VO, 4: NCBITaxon); and Measurements (1: HPO, 2: PRO, 3: CL, 4: NCBITaxon, 5: Uberon, 6: ChEBI).

Acronyms: HPO (Human Phenotype Ontology); Mondo (Monarch Disease Ontology); ChEBI (Chemical Entities of Biological Interest); PRO (Protein Ontology); VO (Vaccine Ontology); NCBITaxon (National Center for Biotechnology Information Taxon Ontology); CL (Cell Ontology); Uberon (Uber-Anatomy Ontology).

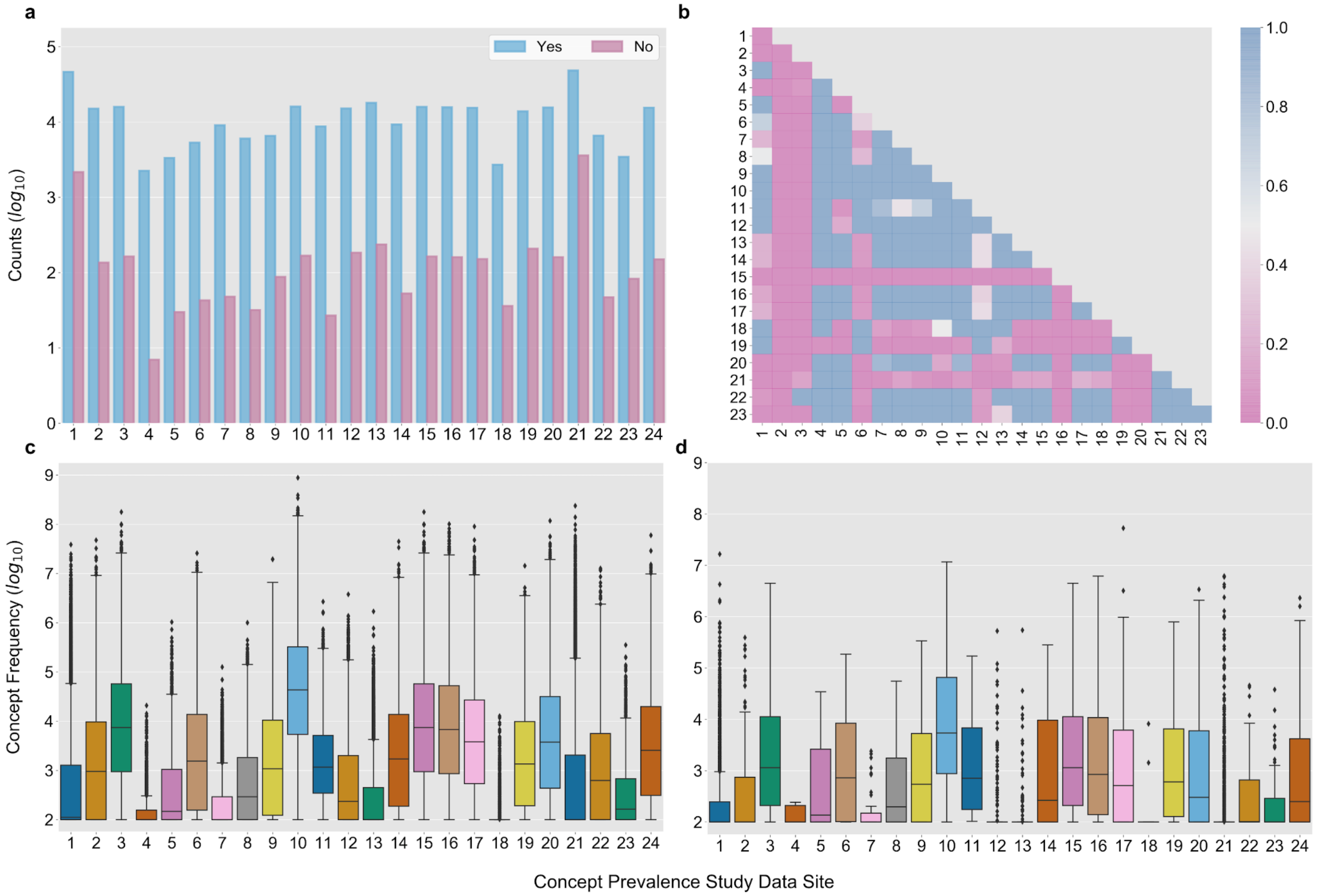

Supplementary Figure 3: Overview of the OMOP2OBO Condition Concepts in the OHDSI Concept Prevalence Data by Coverage Status.

(**A**) This figure presents the counts of OMOP (Observational Medical Outcomes Partnership) condition concepts (log 10 scale) in the Concept Prevalence Study data by site (1-24) and OMOP2OBO mapping set coverage status (“Yes"/”No”). (**B**) This figure visualizes the results of conducting a chi-square test of independence with Yate's correction to assess differences in the proportions of OMOP condition concepts covered by the OMOP2OBO mapping set across the Concept Prevalence Study data sites. The figure presents a heatmap to visualize Bonferroni adjusted p-values for post-hoc tests which confirmed that 32% of the pairwise site comparisons had significantly different coverage of the OMOP2OBO mapping sets (ps<0.001 for all significant comparisons). (**C**) This figure presents the frequency distributions of OMOP condition concepts covered by the OMOP2OBO mapping set (log 10 scale) in the Concept Prevalence Study data by site. (**D**) This figure presents the frequency distributions of OMOP condition concepts not covered by the OMOP2OBO mapping set (log 10 scale) in the Concept Prevalence Study data by site. Figures **C**-**D**: in each boxplot, the box extends from the first to third quartile of the data with a center line used to indicate the median. Whiskers extend from each box by 1.5x the interquartile range and outliers that extend past the whiskers shown as dots. The x-axis labels are numbers which correspond to the Concept Prevalence Study site index: (1) Ajou University Database; (2) IQVIA US Ambulatory Electronic Medical Record; (3) IQVIA Longitudinal Patient Data Australia; (4) IQVIA Disease Analyzer France; (5) IQVIA Disease Analyzer Germany; (6) The Healthcare Cost and Utilization Project Nationwide Inpatient Sample; (7) IQVIA US Hospital Charge Data Master; (8) IBM MarketScan Commercial Database; (9) IBM MarketScan Multi-State Medicaid Database; (10) IBM MarketScan Medicare Supplemental Database; (11) Japan Medical Data Center database; (12) Medical Information Mart for Intensive Care III; (13) Korea National Health Insurance Service/National Sample Cohort; (14) Optum De-Identified Clinformatics Data-Mart-Database—Date of Death; (15) Optum De-Identified Clinformatics Data-Mart-Database—Socio-Economic Status; (16) Optum De-identified Electronic Health Record Dataset; (17) IQVIA US LRxDx Open Claims; (18) Premier Healthcare Database; (19) University of Southern California PScanner; (20) Stanford Medicine Research Data Repository; (21) Tufts Medical Center Database; (22) University of Colorado Anschutz Medical Campus Health Group; (23) Australian Electronic Practice-based Research Network; (24) Columbia University Medical Center Database.

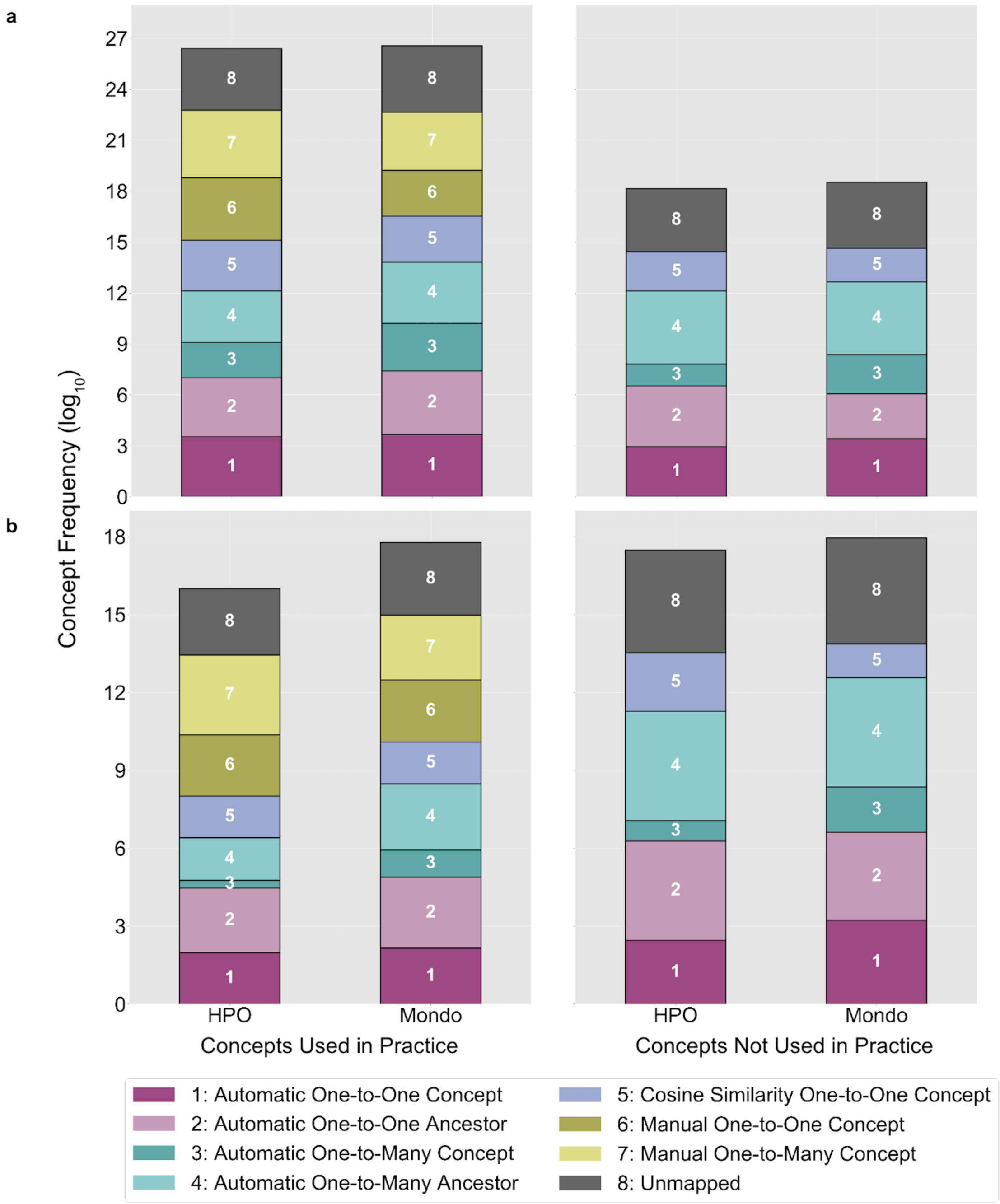

Supplementary Figure 4: Frequency of OMOP2OBO Condition Concepts in the OHDSI Concept Prevalence Data by OBO Foundry Ontology and Data Wave.

(**A**) This figure visualizes the count of Observational Medical Outcomes Partnership (OMOP) condition concepts (log 10 scale) in the OMOP2OBO mapping set that overlapped with Concept Prevalence Study by Open Biological and Biomedical Ontology (OBO) Foundry ontology and (Concepts Used in Practice [all concepts associated with at least one patient and/or visit in the Children's Hospital of Colorado OMOP Database] and Concepts Not Used in Practice [all concepts not used in clinical practice]). (**B**) This figure visualizes the count of OMOP condition concepts (log 10 scale) in the OMOP2OBO mapping set condition concepts that were not present in the Concept Prevalence Study data by OBO Foundry ontology and data wave. The labels on the bars are numbers which correspond to the OMOP2OBO mapping categories: (1) Automatic One-to-One Concept; (2) Automatic One-to-One Ancestor (3) Automatic One-to-Many Concept; (4) Automatic One-to-Many Ancestor; (5) Cosine Similarity One-to-One Concept; (6) Manual One-to-One Concept; (7) Manual One-to-Many Concept; and (8) Unmapped.

Acronyms: HPO (Human Phenotype Ontology); Mondo (Monarch Disease Ontology).

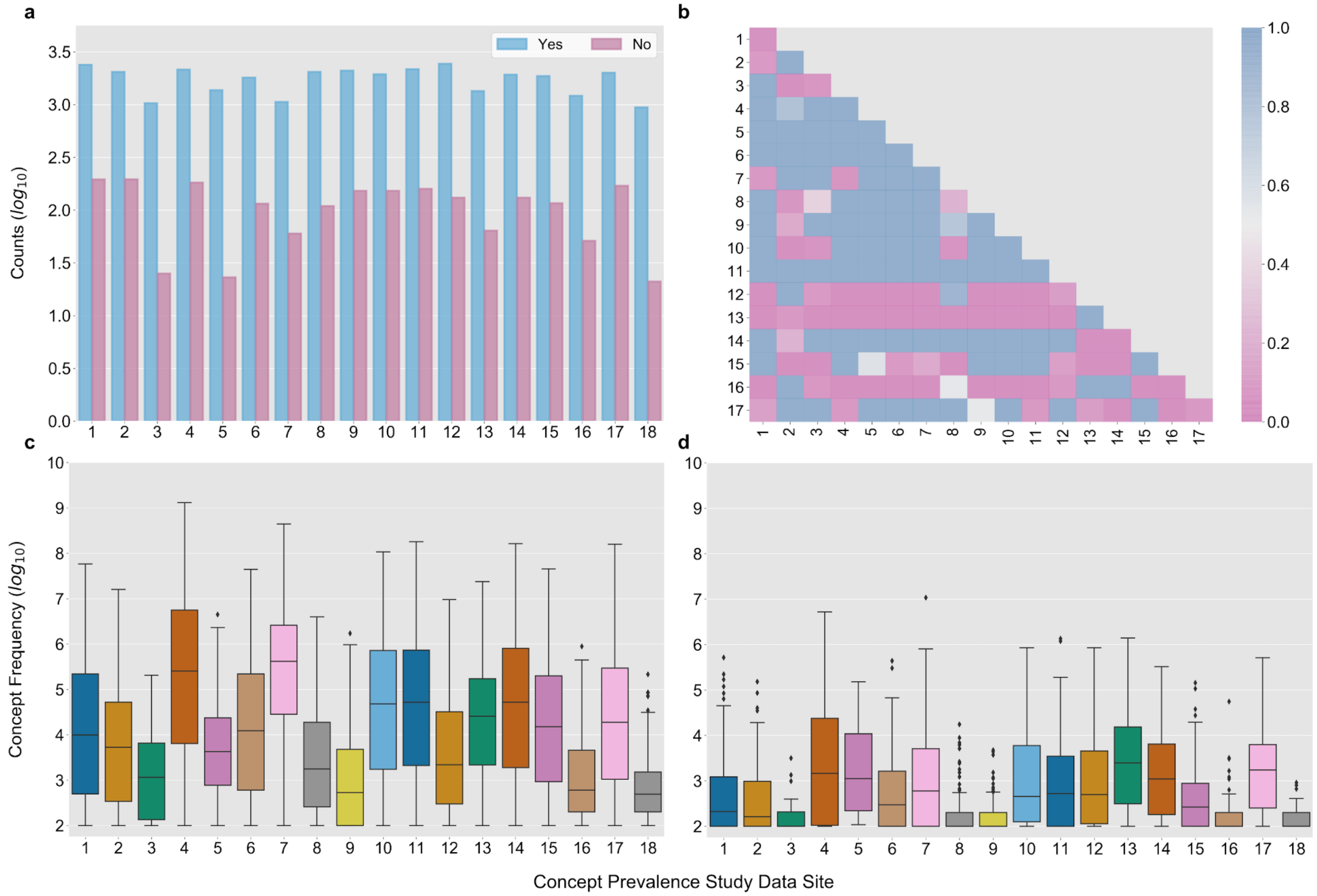

Supplementary Figure 5: Overview of the OMOP2OBO Drug Ingredient Concepts in the OHDSI Concept Prevalence Data by Coverage Status.

(**A**) This figure presents the counts of OMOP (Observational Medical Outcomes Partnership) drug ingredient concepts (log 10 scale) in the Concept Prevalence Study data by site (1-18) and OMOP2OBO mapping set coverage status (“Yes"/”No”). (**B**) This figure visualizes the results of conducting a chi-square test of independence with Yate's correction to assess differences in the proportions of OMOP drug ingredient concepts covered by the OMOP2OBO mapping set across the Concept Prevalence Study data sites. The figure presents a heatmap to visualize Bonferroni adjusted p-values for post-hoc tests which confirmed that 22% of the pairwise site comparisons had significantly different coverage of the OMOP2OBO mapping sets (ps<0.001 for all significant comparisons). (**C**) This figure presents the frequency distributions of OMOP drug ingredient concepts covered by the OMOP2OBO mapping set (log 10 scale) in the Concept Prevalence Study data by site. (**D**) This figure presents the frequency distributions of OMOP drug ingredient concepts not covered by the OMOP2OBO mapping set (log 10 scale) in the Concept Prevalence Study data by site. Figures **C**-**D**: in each boxplot, the box extends from the first to third quartile of the data with a center line used to indicate the median. Whiskers extend from each box by 1.5x the interquartile range and outliers that extend past the whiskers shown as dots. The x-axis labels are numbers which correspond to the Concept Prevalence Study site index: (1) IQVIA US Ambulatory Electronic Medical Record; (2) IQVIA Longitudinal Patient Data Australia; (3) IQVIA Disease Analyzer Germany; (4) IQVIA US Hospital Charge Data Master; (5) IBM MarketScan Commercial Database; (6) IBM MarketScan Multi-State Medicaid Database; (7) IBM MarketScan Medicare Supplemental Database; (8) Japan Medical Data Center database; (9) Optum De-Identified Clinformatics Data-Mart-Database—Socio-Economic Status; (10) Optum De-identified Electronic Health Record Dataset; (11) Optum De-identified Electronic Health Record Dataset; (12) Premier Healthcare Database; (13) University of Southern California PScanner; (14) Stanford Medicine Research Data Repository; (15) Tufts Medical Center Database; (16) University of Colorado Anschutz Medical Campus Health Group; (17) Australian Electronic Practice-based Research Network; (18) Columbia University Medical Center Database.

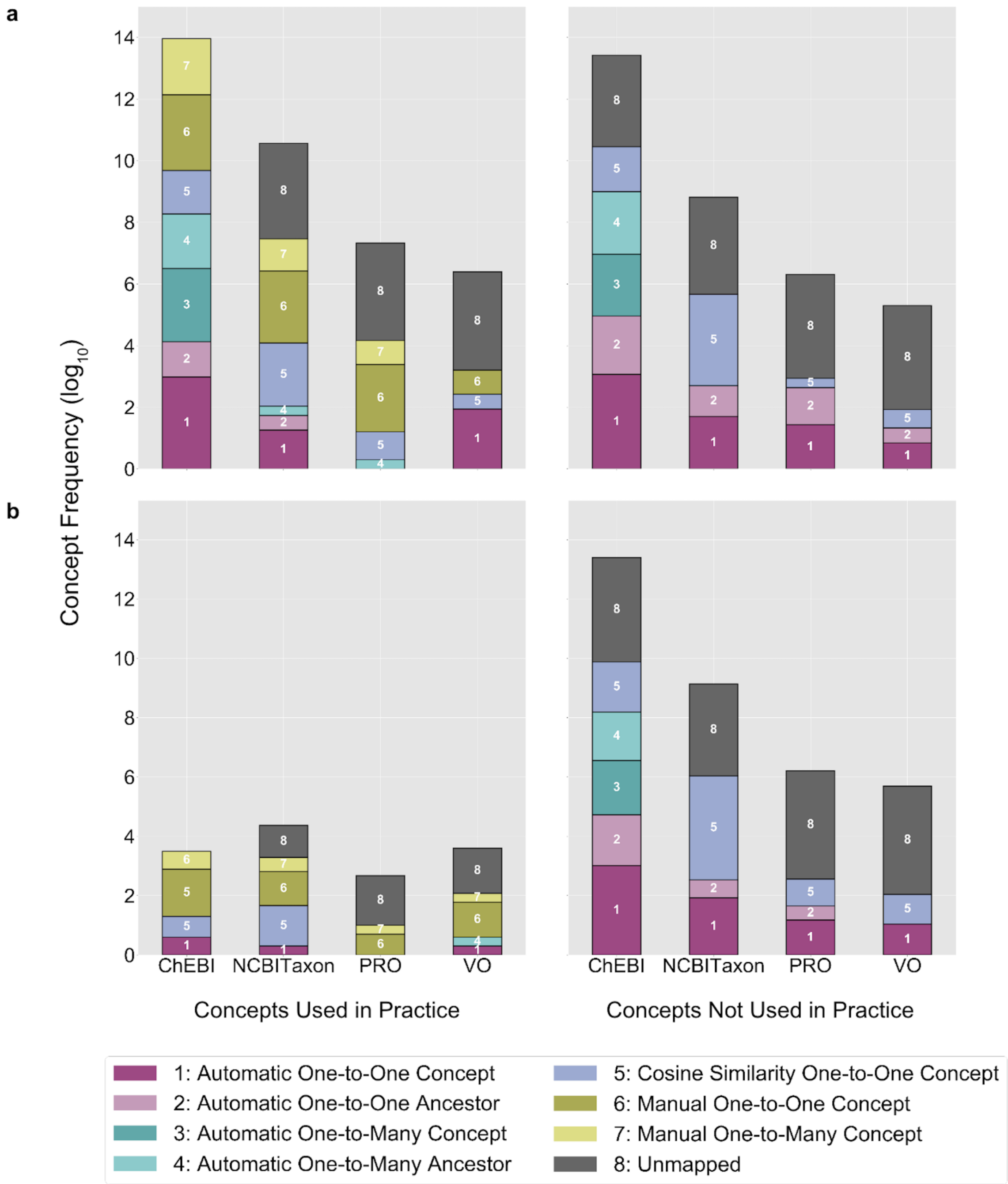

Supplementary Figure 6: Frequency of OMOP2OBO Drug Ingredient Concepts in the OHDSI Concept Prevalence Data by OBO Foundry Ontology and Data Wave.

(**A**) This figure visualizes the count of Observational Medical Outcomes Partnership (OMOP) drug ingredient concepts (log 10 scale) in the OMOP2OBO mapping set that overlapped with concepts in the Concept Prevalence Study by Open Biological and Biomedical Ontology (OBO) Foundry ontology and data wave (Concepts Used in Practice [all concepts associated with at least one patient and/or visit in the Children's Hospital of Colorado OMOP Database] and Concepts Not Used in Practice [all standard OMOP concepts not used in clinical practice]). (**B**) This figure visualizes the count of OMOP drug ingredient concepts (log 10 scale) in the OMOP2OBO mapping set that were not present in the Concept Prevalence Study data by OBO Foundry ontology and data wave. The labels on the bars are numbers which correspond to the OMOP2OBO mapping categories: (1) Automatic One-to-One Concept; (2) Automatic One-to-One Ancestor (3) Automatic One-to-Many Concept; (4) Automatic One-to-Many Ancestor; (5) Cosine Similarity One-to-One Concept; (6) Manual One-to-One Concept; (7) Manual One-to-Many Concept; and (8) Unmapped.

Acronyms: ChEBI (Chemical Entities of Biological Interest); NCBITaxon (National Center for Biotechnology Information Taxon Ontology); PRO (Protein Ontology); VO (Vaccine Ontology).

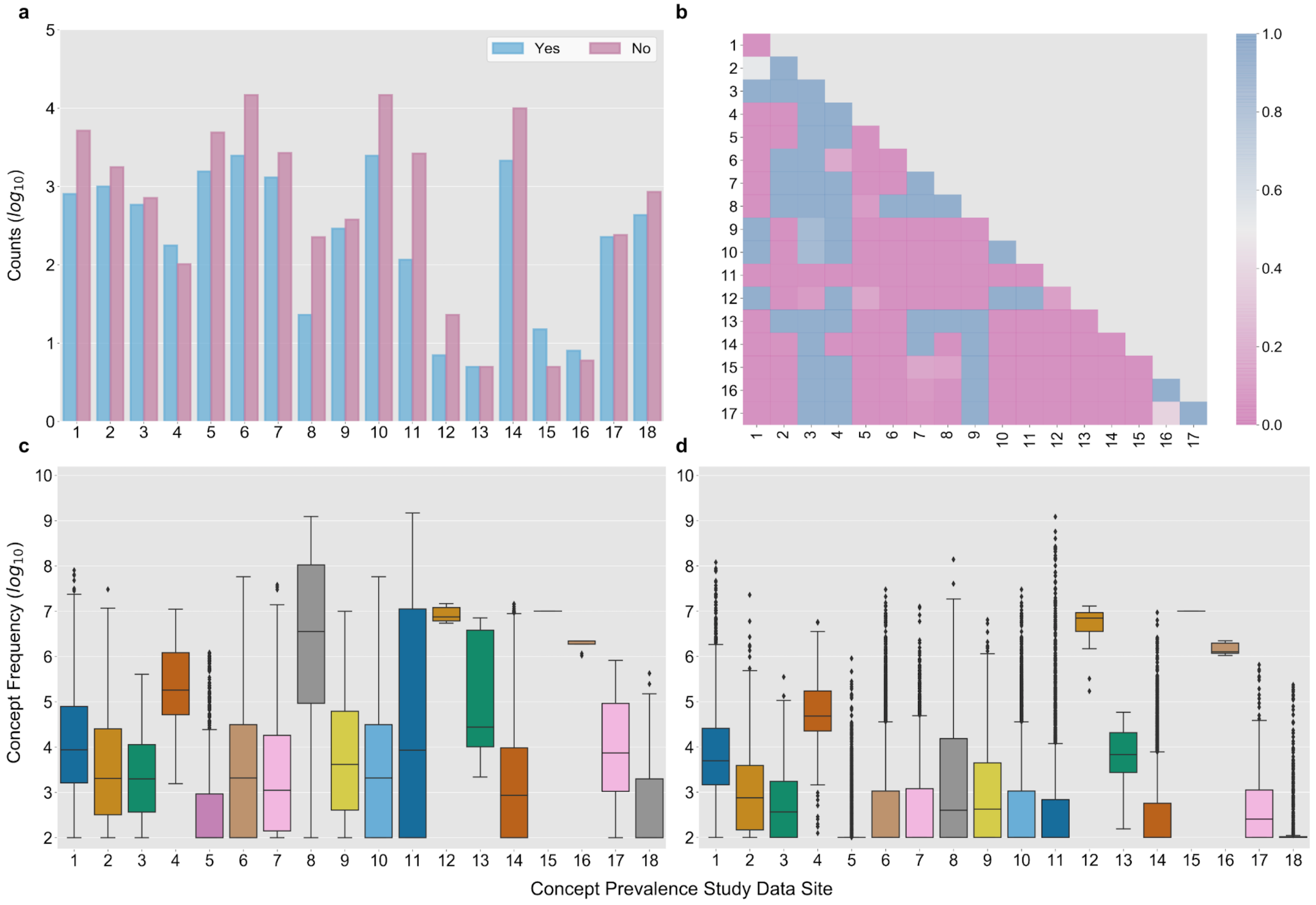

Supplementary Figure 7: Overview of the OMOP2OBO Measurement Concepts in the OHDSI Concept Prevalence Data by Coverage Status.

(**A**) This figure presents the counts of OMOP (Observational Medical Outcomes Partnership) measurement concepts (log 10 scale) in the Concept Prevalence Study data by site (1-18) and OMOP2OBO mapping set coverage status ("Yes"/"No"). (**B**) This figure visualizes the results of conducting a chi-square test of independence with Yate's correction to assess differences in the proportions of OMOP measurement concepts covered by the OMOP2OBO mapping set across the Concept Prevalence Study data sites. The figure presents a heatmap to visualize Bonferroni adjusted p-values for post-hoc tests which confirmed that 56% of the pairwise site comparisons had significantly different coverage of the OMOP2OBO mapping sets (ps<0.001 for all significant comparisons). (**C**) This figure presents the frequency distributions of OMOP measurement concepts covered by the OMOP2OBO mapping set (log 10 scale) in the Concept Prevalence Study data by site. (**D**) This figure presents the frequency distributions of OMOP measurement concepts not covered by the OMOP2OBO mapping set (log 10 scale) in the Concept Prevalence Study data by site. Figures **C**-**D**: in each boxplot, the box extends from the first to third quartile of the data with a center line used to indicate the median. Whiskers extend from each box by 1.5x the interquartile range and outliers that extend past the whiskers shown as dots. The x-axis labels are numbers which correspond to the Concept Prevalence Study site index: (1) IQVIA US Ambulatory Electronic Medical Record; (2) IQVIA Longitudinal Patient Data Australia; (3) IQVIA Disease Analyzer France; (4) IQVIA Disease Analyzer Germany; (5) IBM MarketScan Commercial Database; (6) IBM MarketScan Medicare Supplemental Database; (7) Japan Medical Data Center database; (8) Medical Information Mart for Intensive Care III; (9) Korea National Health Insurance Service/National Sample Cohort; (10) Optum De-Identified Clinformatics Data-Mart-Database—Date of Death; (11) Optum De-Identified Clinformatics Data-Mart-Database—Socio-Economic Status; (12) Optum De-identified Electronic Health Record Dataset; (13) Premier Healthcare Database; (14) University of Southern California PScanner; (15) Stanford Medicine Research Data Repository; (16) University of Colorado Anschutz Medical Campus Health Group; (17) Australian Electronic Practice-based Research Network; (18) Columbia University Medical Center Database.

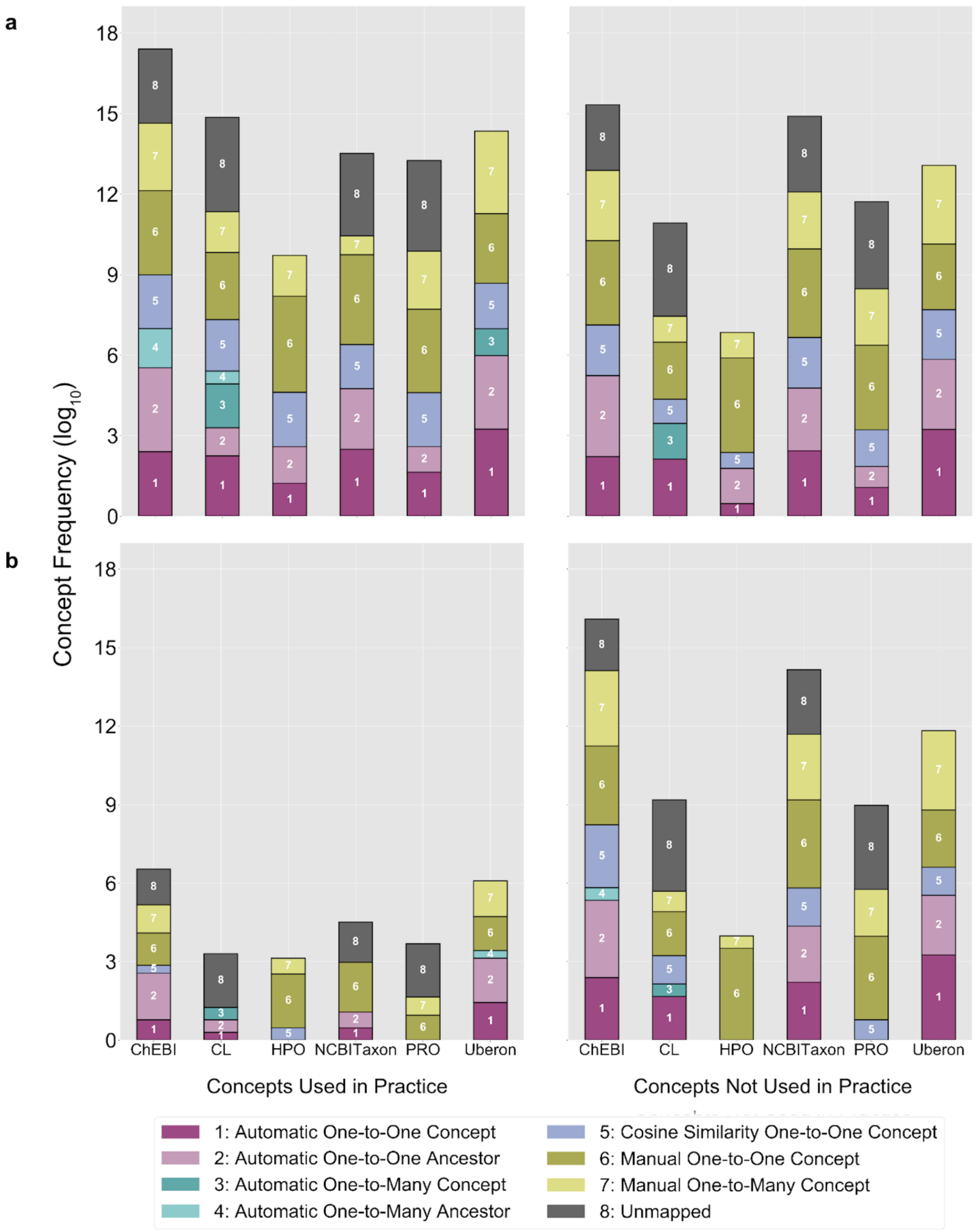

Supplementary Figure 8: Frequency of OMOP2OBO Measurement Concepts in the OHDSI Concept Prevalence Data by OBO Foundry Ontology and Data Wave.

(**A**) This figure visualizes the count of Observational Medical Outcomes Partnership (OMOP) measurement concepts (log 10 scale) in the OMOP2OBO mapping set that overlapped with concepts in the Concept Prevalence Study by Open Biological and Biomedical Ontology (OBO) Foundry ontology and data wave (Concepts Used in Practice [all concepts associated with at least one patient and/or visit in the Children's Hospital of Colorado OMOP Database] and Concepts Not Used in Practice [all concepts not used in clinical practice]). (**B**) This figure visualizes the count of OMOP measurement concepts (log 10 scale) in the OMOP2OBO mapping set that were not present in the Concept Prevalence Study data by OBO Foundry ontology and data wave. The labels on the bars are numbers which correspond to the OMOP2OBO mapping categories: (1) Automatic One-to-One Concept; (2) Automatic One-to-One Ancestor; (3) Automatic One-to-Many Concept; (4) Automatic One-to-Many Ancestor; (5) Cosine Similarity One-to-One Concept; (6) Manual One-to-One Concept; (7) Manual One-to-Many Concept; and (8) Unmapped.

Acronyms: ChEBI (Chemical Entities of Biological Interest); CL (Cell Ontology); HPO (Human Phenotype Ontology); NCBITaxon (National Center for Biotechnology Information Taxon Ontology); PRO (Protein Ontology); Uberon (Uber-Anatomy Ontology).

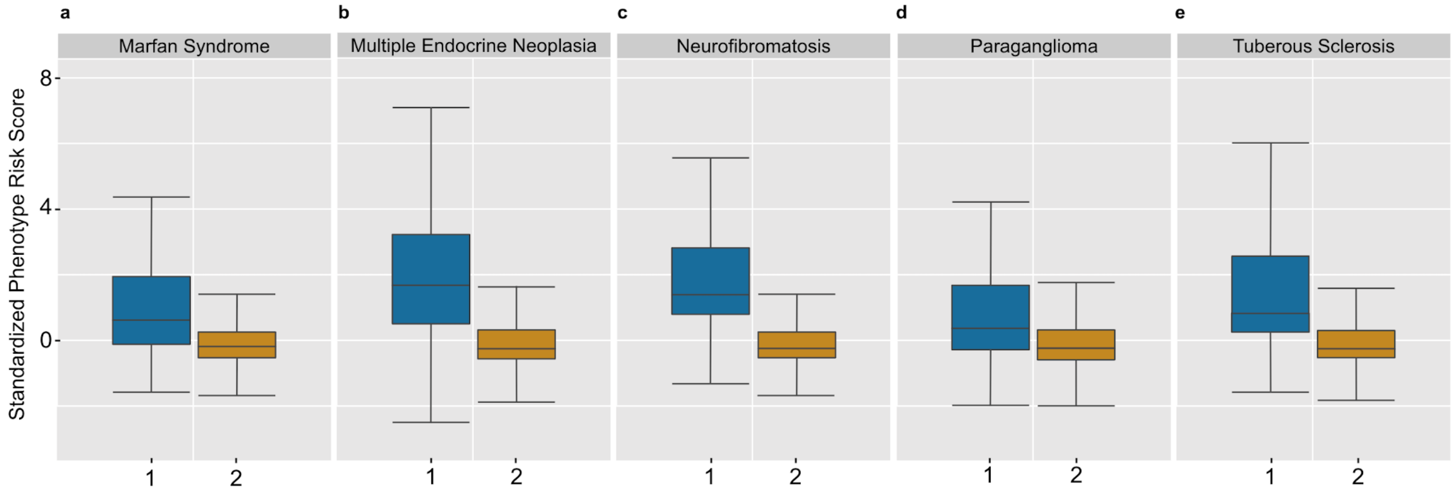

Supplementary Figure 9: Standardized Phenotype Risk Scores (PheRS) by Disease for Cases and Controls.

The Phenotype Risk Score (PheRS) is a measure used to identify patients with phenotypic features that are clinically similar to Online Mendelian Inheritance in Man (OMIM) Mendelian profiles but who lack formal diagnosis and has demonstrated utility for identifying underdiagnosed rare disease patients using only electronic health record data. The standardized PheRS was applied to five diseases (Figures **A-E**) known to be caused by pathogenic genetic mutations in 11 American College of Medical Genetics and Genomics secondary finding genes (listed by disease below). In this figure, boxplots of the PheRS are used to illustrate differences between cases and controls for each of the five diseases using data from the *All of Us* Research Program. To determine if the PheRSs were significantly higher for cases than controls, one-sided Wilcoxon rank sum tests were performed for each disease. Results confirmed that cases had significantly higher PheRS than controls for all examined diseases ($p<0.001$ across all diseases), which included: (**A**) Marfan syndrome (*FBN1*, *TGFBR1*); (**B**) multiple endocrine neoplasia related to (*MEN1*, *RET*); (**C**) neurofibromatosis (*NF2*); (**D**) paragangliomas (related to succinate dehydrogenase genes: *SDHAF2*, *SDHB*, *SDHC*, *SDHD*); and (**E**) tuberous sclerosis complex (*TSC1*, *TSC2*). In each boxplot, the box extends from the first to third quartile of the data with a center line used to indicate the median. Whiskers extend from each box by 1.5x the interquartile range and outliers that extend past the whiskers shown as dots. The x-axis labels are numbers which correspond to control (blue) and case (yellow) patients.

Supplementary Table 8: Descriptive Statistics by Disease for Cases and Controls.

	Marfan Syndrome	Multiple Endocrine Neoplasia	Neurofibromatosis	Paraganglioma	Tuberous Sclerosis
Cases					
Patient Count	131	86	255	105	38
Standardized PheRS[a]					
Mean	1.136	2.147	1.968	1.072	1.317
Median	0.616	1.673	1.381	0.378	0.824
Standard Deviation	2.020	2.375	1.981	2.308	1.811
Range (min, max)	-3.326, 11.521	-2.512, 11.402	-1.305, 10.767	-1.970, 10.249	-1.578, 6.003
Controls					
Patient Count	63,086	72,150	65,256	68,552	58,555
Standardized PheRS[a]					
Mean	-0.013	-0.004	-0.006	-0.002	-0.009
Median	-0.186	-0.245	-0.234	-0.239	-0.264
Standard Deviation	0.949	0.996	0.993	1.001	0.989
Range (min, max)	-12.476, 7.366	-12.305, 11.213	-9.393, 13.595	-9.919, 13.539	-10.544, 23.098

[a]The standardized PheRS is derived by subtracting the normalized raw scores by the mean and dividing by the standard deviation.
Acronyms: PheRS (Phenotype Risk Score).
