# Supplementary material for: Ontologizing health systems data at scale: making translational discovery a reality
Source: NPJ Digit Med. 2023 May 19;6:89. doi: 10.1038/s41746-023-00830-x (PMC10196319; doi:10.1038/s41746-023-00830-x)
Supplement: Supplementary file 2 — Reporting Summary [file 41746_2023_830_MOESM2_ESM.pdf]

nature portfolio

Corresponding author(s): Tiffany J. Callahan

Last updated by author(s): 02/01/2023

Reporting Summary

Nature Portfolio wishes to improve the reproducibility of the work that we publish. This form provides structure for consistency and transparency in reporting. For further information on Nature Portfolio policies, see our Editorial Policies and the Editorial Policy Checklist.

Statistics

For all statistical analyses, confirm that the following items are present in the figure legend, table legend, main text, or Methods section.

n/a	Confirmed	
☐	☒	The exact sample size (*n*) for each experimental group/condition, given as a discrete number and unit of measurement
☐	☒	A statement on whether measurements were taken from distinct samples or whether the same sample was measured repeatedly
☐	☒	The statistical test(s) used AND whether they are one- or two-sided<br>*Only common tests should be described solely by name; describe more complex techniques in the Methods section.*
☐	☒	A description of all covariates tested
☐	☒	A description of any assumptions or corrections, such as tests of normality and adjustment for multiple comparisons
☐	☒	A full description of the statistical parameters including central tendency (e.g. means) or other basic estimates (e.g. regression coefficient) AND variation (e.g. standard deviation) or associated estimates of uncertainty (e.g. confidence intervals)
☐	☒	For null hypothesis testing, the test statistic (e.g. *F*, *t*, *r*) with confidence intervals, effect sizes, degrees of freedom and *P* value noted<br>*Give P values as exact values whenever suitable.*
☒	☐	For Bayesian analysis, information on the choice of priors and Markov chain Monte Carlo settings
☒	☐	For hierarchical and complex designs, identification of the appropriate level for tests and full reporting of outcomes
☐	☒	Estimates of effect sizes (e.g. Cohen's *d*, Pearson's *r*), indicating how they were calculated

Our web collection on statistics for biologists contains articles on many of the points above.

Software and code

Policy information about availability of computer code

Data collection

OMOP2OBO Mapping Algorithm Data: The algorithm relies on data from the UMLS and vocabulary data from the OMOP CDM. Two tables from the UMLS (MRCONSO and MRSTY [2020AA version]) are required. These data were downloaded directly from the UMLS (https://www.nlm.nih.gov/research/umls/licensedcontent/umlsarchives04.html). Similar to the data within the UMLS, some of the vocabularies within the OMOP CDM may require a license to use and this use may depend upon the vocabulary or interest and the user’s location. All users should read the licensing agreements for both resources and consult their institution before developing new mappings.

Building OMOP2OBO Mappings: The OMOP2OBO mappings were created using a de-identified non-human subjects research approved pediatric dataset from the Children’s Hospital of Colorado normalized to the OMOP CDM (no PHI or patient-level data was included or utilized for this work). The concepts themselves can be obtained directly through the mapping files we provide and through the Athena web app (as noted in our Data Availability statement). We also utilized publicly available OBO Foundry ontology data, downloaded using OWLTools (https://github.com/owlcollab/owltools). The mappings were evaluated using data from the All of Us Research Hub (which were analyzed by an authorized researcher and member of the All of Us Research Program Team using the Researcher Workbench) and data from the Concept Prevalence Study, which was provided by the study lead AO, did not contain any patient-level data (references 1-4).

References
1. Ostropolets, A., Ryan, P. B. & Hripcsak, G. OHDSI Network Study: Concept Prevalence. https://forums.ohdsi.org/t/network-study-concept-prevalence/6562 (2019).
2. Ostropolets, A., Ryan, P. & Hripcsak, G. OHDSI Network Study: Concept Prevalence. https://github.com/ohdsi-studies/ConceptPrevalence (2020).
3. Ostropolets, A., Ryan, P. & Hripcsak, G. Concept Prevalence Study Protocol. https://github.com/ohdsi-studies/ConceptPrevalence/blob/master/extras/ConceptPrevalenceStudyProtocol_v1.0.docx (2020).

4. Ostropolets, A., Ryan, P. & Hripcsak, G. Phenotyping in distributed data networks: selecting the right codes for the right patients. AMIA Annu. Symp. Proc. (2022).

Data analysis

OMOP2OBO was developed using Python 3.6.2 on a single machine with 8 cores and 16GB of RAM. All code and project information are publicly available and detailed on GitHub (https://github.com/callahantiff/OMOP2OBO). The OMOP2OBO (v1.0) mappings are publicly available from Zenodo (see Data Availability Section for URLs). The OMOP2OBO Mapping Dashboard was built with R (v4.2.1) using Rmarkdown (v2.14) and flexdashboard (v0.5.2).

Descriptive and inferential statistics were performed to evaluate the data available for mapping and the OMOP2OBO mapping set. Chi-squared tests of independence with Yate's correction were used to: (I) assess differences in the proportions of metadata available from each OBO Foundry ontology; and (II) assess differences in the proportions of mapped concepts between OHDSI Concept Prevalence sites. Post-hoc tests using Bonferroni adjustment to correct for multiple comparisons were performed for significant omnibus tests. Analyses were performed in Jupyter Notebooks (v6.1.6) using the scipy (v1.4.1), statsmodels (v0.12.1), statistics (v1.0.3.5), and numpy (v.1.18.1) libraries. Visualizations were created using matplotlib (v.3.3.2). The Clinical Utility evaluation was performed in the AoU Researcher Workbench using R (v4.1.2) and Python (v3.7). Analyses were performed on a machine with 16 CPUs and 60GB of memory.

For manuscripts utilizing custom algorithms or software that are central to the research but not yet described in published literature, software must be made available to editors and reviewers. We strongly encourage code deposition in a community repository (e.g. GitHub). See the Nature Portfolio guidelines for submitting code & software for further information.

Data

Policy information about availability of data

All manuscripts must include a data availability statement. This statement should provide the following information, where applicable:

- Accession codes, unique identifiers, or web links for publicly available datasets
- A description of any restrictions on data availability
- For clinical datasets or third party data, please ensure that the statement adheres to our policy

All data are made available via URLs (if publicly available) or by providing details as to how the data can be obtained using GitHub or through the All of Us Research program. Supplementary Tables 2 and 3 list the resources used by the OMOP2OBO algorithm. The MRCONSO and MRSTY tables (2020AA) require a license and are available through the UMLS (https://www.nlm.nih.gov/research/umls/licensedcontent/umlsknowledgesources.html). The data used to build and validate the OMOP2OBO mappings (v1) are described in Supplemental Table 3. The OMOP concepts are available for download through Athena (https://athena.ohdsi.org/). The UMLS data requires a license to use (https://www.nlm.nih.gov/research/umls/licensedcontent/umlsarchives04.html) and some of the OMOP CDM vocabularies also require a license. All users should read the licensing agreements for both resources and consult their institution before developing new mappings.
The OBO Foundry ontologies are publicly available (https://obofoundry.org/). The OMOP2OBO (v1.0) mappings are publicly available and can be downloaded from Zenodo: Conditions (https://doi.org/10.5281/zenodo.6774363); Drugs (https://doi.org/10.5281/zenodo.6774401); and Measurements (https://doi.org/10.5281/zenodo.6774443).

Human research participants

Policy information about studies involving human research participants and Sex and Gender in Research.

Reporting on sex and gender

Sex and gender were not considered as part of our studies or performed analyses. No gender- or sex-specific comparisons were made and no tests were performed that examined the impact of sex or gender.

Population characteristics

The majority of the analyses only considered the total number of clinical vocabulary and ontology concepts at an aggregated level (hospital or research site). For the Clinical Utility evaluation, which was the only component of the study that involved patient data, only one test was performed, which compared phenotype risk scores (PheRS) across five diseases using the All of Us Research Workbench. The PheRS is calculated using a "weighted aggregation of genetically-related phenotypes, analogous to the genetic risk score approach for analyzing multiple variants against a single phenotype" (PMID:29590070). The variables considered when calculating PheRS were limited to ontology concepts.

Recruitment

N/A. Participants were not recruited for the current work. Our work only involved secondary data analysis.

Ethics oversight

Three clinical datasets were utilized: (1) CHCO pediatric data; (2) Concept Prevalence data; and (3) All of Us Research program data. The ethics involved with each data source are described below.

CHCO DATA: Use of the pediatric data was approved by the Colorado Multiple Institutional Review Board (#15-0445), which was determined to be non-human subjects and the data analyzed for this work contained no patient-level data. These data were obtained from a de-identified database that was determined by the Colorado Multiple Institutional Review Board to be non-human subjects (#15-0445). Due to the broad scope of the projects approved to be performed on this database, a Waiver of consent was obtained as it was not practical to obtain consent from all patients.

CONCEPT PREVALENCE DATA: No IRB approval was required for the Concept Prevalence data as it contained no patient-level data. The Concept Prevalence study provides data on the frequency of OMOP concept usage in clinical practice across several independent sites in the OHDSI network.

ALL OF US DATA: All analyses were performed in the All of Us Researcher Workbench by an authorized researcher (CZ). Informed consent is obtained from all participants who enroll in the All of Us Research program (PMID:34115137). Because the authors were not directly involved with the participants and all data were de-identified, the use of these data was exempt from institutional review. For additional details, see “Do I need my project reviewed by the All of Us Institutional

Review Board (IRB) in order to access this data using the Researcher Workbench?" (https://www.researchallofus.org/frequently-asked-questions/#workbench-faqs).

Note that full information on the approval of the study protocol must also be provided in the manuscript.

Field-specific reporting

Please select the one below that is the best fit for your research. If you are not sure, read the appropriate sections before making your selection.

☒ Life sciences ☐ Behavioural & social sciences ☐ Ecological, evolutionary & environmental sciences

For a reference copy of the document with all sections, see nature.com/documents/nr-reporting-summary-flat.pdf

Life sciences study design

All studies must disclose on these points even when the disclosure is negative.

Sample size	No sample size calculations were performed. All available data were used and prior to performing any analyses, a senior biostatistcian verified the data to ensure we had sufficient sample size for the planned analyses.
Data exclusions	No data were purposefully excluded.
Replication	N/A. It was not necessary to replicate any of the analyses performed in this work.
Randomization	N/A. Participants were not recruited for the current work. Our work only involved secondary data analysis.
Blinding	N/A. Blinding was not relevant to our study as it was not pertinent to any of the planned analyses or evaluation tasks.

Reporting for specific materials, systems and methods

We require information from authors about some types of materials, experimental systems and methods used in many studies. Here, indicate whether each material, system or method listed is relevant to your study. If you are not sure if a list item applies to your research, read the appropriate section before selecting a response.

Materials & experimental systems

n/a	Involved in the study	
☒	☐	Antibodies
☒	☐	Eukaryotic cell lines
☒	☐	Palaeontology and archaeology
☒	☐	Animals and other organisms
☒	☐	Clinical data
☒	☐	Dual use research of concern

Methods

n/a	Involved in the study	
☒	☐	ChIP-seq
☒	☐	Flow cytometry
☒	☐	MRI-based neuroimaging
